# Association between Protein Intake and Diabetes Complications Risk Following Incident Type 2 Diabetes: The EPIC-Potsdam Study

**DOI:** 10.3390/metabo14030172

**Published:** 2024-03-19

**Authors:** Elif Inan-Eroglu, Olga Kuxhaus, Franziska Jannasch, Daniela V. Nickel, Matthias B. Schulze

**Affiliations:** 1 Department of Molecular Epidemiology, German Institute of Human Nutrition Potsdam-Rehbruecke, 14558 Nuthetal, Germany; elif.inan-eroglu@dife.de (E.I.-E.); olga.kuxhaus@dife.de (O.K.); franziska.jannasch@dife.de (F.J.); daniela.nickel@dife.de (D.V.N.); 2 German Center for Diabetes Research (DZD), 85764 Neuherberg, Germany; 3 Institute of Nutritional Science, University of Potsdam, 14558 Nuthetal, Germany

**Keywords:** protein intake, type 2 diabetes, diabetes complications, animal protein, plant protein

## Abstract

Our knowledge about the connection between protein intake and diabetes-related complications comes largely from studies among those already diagnosed with type 2 diabetes (T2D). However, there is a lack of information on whether changing protein intake after diabetes diagnosis affects complications risk. We aimed to explore the association between protein intake (total, animal, and plant) and vascular complications in incident T2D patients considering pre-diagnosis intake and changes in intake after diagnosis. This prospective cohort study included 1064 participants from the European Prospective Investigation into Cancer and Nutrition (EPIC)-Potsdam cohort who developed T2D during follow-up (physician-verified). Dietary protein intake was measured with a food frequency questionnaire at baseline and follow-up. We included physician-reported incident diabetes complications (myocardial infarction, stroke, nephropathy, and neuropathy). A total of 388 participants developed complications, 82 macrovascular complications, and 343 microvascular complications. Substituting carbohydrates with protein showed a trend towards lower complications risk, although this association was not statistically significant (hazard ratio (HR) for 5% energy (E) substitution: 0.83; 95% confidence intervals (CI): 0.60–1.14). Increasing protein intake at the expense of carbohydrates after diabetes diagnosis was not associated with total and microvascular complications (HR for 5% E change substitution: 0.98; 95% CI: 0.89–1.08 and HR for 5% E change substitution: 1.02; 95% CI: 0.92–1.14, respectively). Replacing carbohydrates with protein did not elevate the risk of diabetes complications in incident T2D cases.

## 1. Introduction

People with T2D face the risk of developing serious complications, including cardiovascular disease (CVD), neuropathy, nephropathy, retinopathy, and cancer, collectively contributing to a potential reduction in life expectancy of almost a decade [1,2]. Additionally, costs associated with diabetes start to increase at least 8 years prior to diagnosis and escalate more rapidly as the time of diagnosis approaches and immediately after the diagnosis [3].

Evidence suggests that dietary factors may influence the risk of T2D and also of its complications [4]. Cohort studies found that animal-sourced foods with high protein were positively related to T2D risk [5,6], whereas plant-based high-protein foods were associated with lower risk of T2D [7,8]. Cohort studies also evaluated the association between protein intake and T2D risk; for example, we previously observed in the European Prospective Investigation into Cancer and Nutrition (EPIC)-Potsdam cohort that substituting carbohydrates for protein was inversely related to diabetes risk [9]. A recent meta-analysis examining the dose-response relationship between dietary protein intake and T2D risk concluded that a 5% increase in energy from dietary total and animal protein intake was related to a 9% and 12% higher risk of T2D respectively [10]. Still, an umbrella review of systematic reviews of cohort studies concluded that elevated total protein consumption might be linked to an increased risk of T2D, yet the available evidence does not support that consuming more animal protein leads to a higher risk or that consuming more plant protein leads to a lower risk of T2D [11]. Protein intake also holds particular interest because a high dietary intake was traditionally implicated as a potential harm to renal function [12,13]. In individuals with diabetes, a higher intake of protein was positively associated with a greater decline in glomerular filtration rate (GFR), which is a measure of renal function [14]. Beyond its potential role in promoting diabetic nephropathy, elevated protein intake may also be associated with an increased risk of CVD; however, conflicting evidence from studies precludes the establishment of a definitive association [15,16]. In contrast to the notion that protein intake increases complications risk, high protein intake in T2D patients results in only minor increases in blood glucose, and protein requires less insulin for its metabolism, which in turn reduces insulin-induced lipogenesis and improves blood lipids [17,18,19].

Previous studies on protein intake and diabetes-related vascular complications were based on people with prevalent T2D and were limited to a single complication [20,21,22,23]. Thus, data to support recommendations for adapting protein intake after a diagnosis of T2D as part of medical nutrition therapy are scarce. The aim of this study is to investigate the association of changing protein intake, including total, animal, and plant-based protein, after a T2D diagnosis with the risk of microvascular and macrovascular complications.

## 2. Methods

### 2.1. Study Population

This research was conducted using the EPIC-Potsdam study, a population-based prospective cohort study established to investigate the role of diet in chronic disease occurrence. Participants were recruited from 1994 to 1998 in Potsdam, Germany, and the surrounding geographic communities based on random registry sampling. Overall, 27,548 participants were enrolled, 16,644 women aged 35–64 years and 10,904 men aged 40–64 years [24]. Follow-up questionnaires were implemented every 2–3 years, with response rates exceeding 90% for all follow-up rounds. Detailed information about recruitment and follow-up procedures has been reported elsewhere [25,26]. All participants provided informed consent before enrolment and ethical approval was obtained from the Medical Society of the State of Brandenburg, Germany.

**Figure 1:**
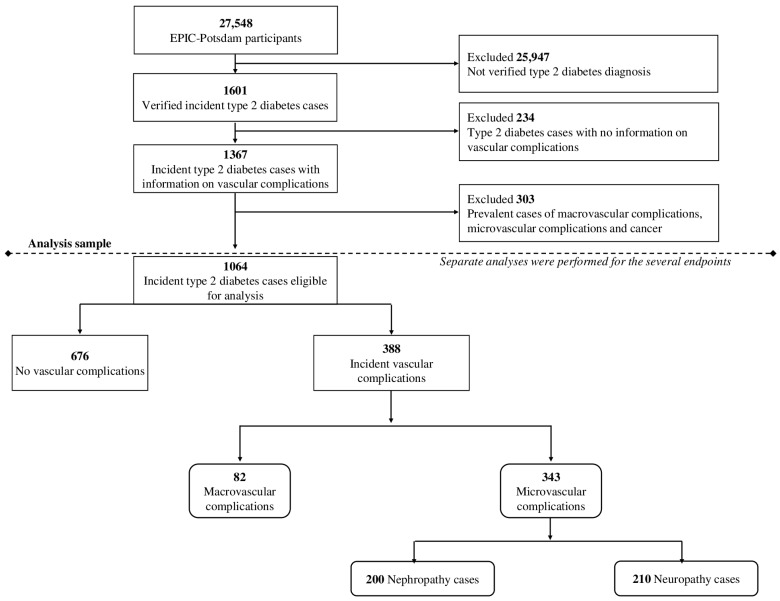
Flowchart of participants.

For this study, we considered individuals who were newly diagnosed with T2D between the time of recruitment and December 2009. We identified a total of 1601 cases of newly diagnosed T2D, confirmed by physicians. We excluded 234 participants who lacked information about vascular complications. Additionally, we removed 303 participants who had been diagnosed with CVD, T2D complications, or cancer before their diabetes diagnosis (n = 1064) (Figure 1).

For an additional analysis involving prevalent cases of T2D, we included 1123 participants who already had T2D at baseline. We used their baseline dietary information to represent protein intake after their diagnosis. We also included participants who were diagnosed with T2D between baseline and the follow-up3 as prevalent T2D cases to increase the size of the sample for analysis. For these individuals, we used their dietary information at the follow-up3 to reflect their diet after diagnosis.

### 2.2. Assessment of Protein Intake

At baseline (between 1994 and 1998) and the third follow-up (between 2001 and 2005), all participants were asked to fill out a semi-quantitative food frequency questionnaire (FFQ), which evaluated the typical frequency of consumption and the portion size for 149 different foods consumed during the 12 months before examination at baseline and 105 different foods consumed during the 12 months before third follow-up. The reproducibility and validity of the FFQ were previously reported [27]. Assessment of protein intake was validated with 24-h urinary nitrogen excretion, which is considered the gold standard [27]. The frequency of consumption was measured using ten categories, ranging from ‘never’ to ‘five times per day or more.’ Photographs of standard portion sizes were used to estimate portion sizes. We used information on the frequency of intake and portion size to calculate the average amount of each food item consumed in grams per day. Protein intake was calculated as g/d based on the German Food Code and Nutrient Database, version II.3 [28].

### 2.3. Ascertainment of Type 2 Diabetes and Vascular Complications

Incident cases of T2D were primarily identified through follow-up self-report questionnaires, where individuals reported disease occurrence, relevant medication use, or dietary treatment. Additional information was collected from death certificates or clinical record linkage. The physicians responsible for participant care validated all potential cases of diabetes. We only included physician-verified T2D cases (with ICD-10 code E11) with diagnosis dates after recruitment.

We obtained information on incident diabetes-related complications through standardised forms (details on the most recent clinic visit, encompassing the instances and dates of vascular complications) sent to treating physicians in 2014, irrespective of participants’ vital status. Macrovascular complications incidence was identified during regular follow-ups of participants using the same procedure as identifying incident T2D cases.

Microvascular complications included diabetic kidney disease (ICD-10 E11.2; unspecified diabetes-related nephropathy, renal replacement therapy, or albuminuria), retinopathy (ICD-10 E11.3; proliferative, non-proliferative retinopathy, or blindness) and neuropathy (ICD-10 E11.4; unspecified diabetes-related peripheral neuropathy, amputation, loss of sensation or diabetic foot syndrome). Macrovascular complications were defined as myocardial infarction (ICD-10 I21) or stroke (ICD-10 I60, I61, I63, I64).

### 2.4. Assessment of Covariates

General characteristics of sociodemographics and lifestyle were assessed using self-administered and interviewer-based questionnaires [24]. Total energy intake (kcal/day), alcohol, fat, fibre, polyunsaturated fatty acids/saturated fatty acids (PUFA/SFA) ratio, cholesterol, magnesium (Mg), and vitamin E intake were derived from the self-reported dietary intake assessed by FFQ [27]. Educational attainment was categorised as no vocational training, technical college and university. Physical activity was defined as the mean time (h/w) spent on sports, biking and gardening. At recruitment, weight and height were assessed by trained interviewers following standard protocols [29]. Self-reported weight was obtained through follow-up questionnaires. Body mass index (kg/m^2^) was calculated as weight (kg) divided by the square of height (m) [30]. Smoking status was categorised as never smoker, former smoker, or current smoker. Information on family history of myocardial infarction and stroke, prevalent hypertension (defined at baseline as systolic BP ≥ 140 mmHg, diastolic BP ≥ 90 mmHg, antihypertensive medication use or prior diagnosis of hypertension) and dyslipidaemia (defined at baseline as lipid-lowering medication use or prior diagnosis of hypertriacylglycerolaemia or hypercholesterolaemia), antidiabetic, lipid-lowering and antihypertensive medication was also collected. Follow-up assessment of hypertension and dyslipidaemia was based on self-reports or medication.

### 2.5. Statistical Analyses

We handled missing values using multiple imputations (m = 10) by chained equations, with imputation models specified for each variable with missing values separately. The variables were sorted by the amount of missing values. We only imputed missing values from completed follow-up rounds. For continuous variables, Box-Cox transformation (for non-normally distributed variables) and the predictive mean matching method were performed.

When evaluating associations of diet before diabetes diagnosis and complications risk, we used Cox proportional hazard models to estimate HRs for the effect of isoenergetic substitution of total, animal and plant protein (per 5% energy (5% E), adjusted for total energy intake by using multivariate nutrient density model) for carbohydrates on diabetes complications as well as for the effect between isoenergetic substitution of animal protein for plant protein on diabetes complications. Age was the underlying timescale, with entry time as age at diabetes diagnosis and exit time as age at event or censoring. We considered dietary intake from the baseline FFQ for all dietary exposures, while the most recent information before diabetes diagnosis was used for other covariates for adjustment. The first model was adjusted for sex (categorical; female, male), total energy intake (continuous; kcal), fat (continuous; %E) and alcohol intake (continuous; %E). Model 2 further included education (categorical; no vocational training/vocational training, technical college, university), physical activity (continuous; h/week), BMI (continuous; kg/m^2^), smoking status (categorical; never-smoker, former smoker, current smoker) and duration (continuous; years), family history of myocardial infarction and stroke, prevalent conditions of hypertension and dyslipidaemia, medication use (antidiabetic, lipid-lowering and antihypertension) and the duration between diet assessment and T2D diagnosis (continuous; years). Model 3 was additionally adjusted for diet-related variables, including fibre intake (continuous; g/day), PUFA/SFA ratio (continuous), cholesterol intake (continuous; mg/day), Mg intake (continuous; mg/day) and vitamin E intake (continuous; mg/day). Macronutrient intakes were entered into the model per 5% E. Total energy intake was entered into the model to keep energy intake constant, which is essential for creating an isocaloric model.

We also evaluated associations with total and microvascular complications for changes in diet from baseline to post-diagnosis. For this analysis, we restricted the study sample to those participants who had diabetes diagnosis between baseline and follow-up3, when the diet was assessed for a second time, and complication diagnosis after follow-up3 (imputed datasets involve 662 to 664 participants). We conducted Cox proportional hazard models using the difference in protein intake between follow-up3 and baseline as main exposure, with the first model also including baseline protein intake, energy intake (baseline and follow-up), fat intake (baseline and follow-up), alcohol intake (baseline and follow-up), age, sex, duration between baseline and follow-up3 as well as duration between T2D diagnosis and follow-up3. The second model included education, physical activity (baseline and follow-up), BMI (baseline and follow-up), smoking status and duration (baseline and follow-up), family history of diabetes, myocardial infarction and stroke, prevalent conditions of hypertension and dyslipidaemia and medication use (antidiabetic, lipid-lowering and antihypertension). Model 3 was additionally adjusted for diet-related variables (baseline and follow-up), including the PUFA/SFA ratio and intake of fibre, cholesterol, magnesium, and vitamin E. This model reflects an isocaloric increase in the percentage of protein that contributes to energy at the expense of carbohydrates. While entry time in Cox models was age at diabetes diagnosis, we repeated the analysis by considering the follow-up3 assessment date (after diagnosis) as entry time to evaluate the robustness of our results. We also stratified the participants into low and high-protein intake groups based on the median value of baseline protein consumption.

For the additional analysis of the effect of isoenergetic substitution of total protein (per 5% E) for carbohydrates on myocardial infarction, stroke and total CVD among participants with prevalent diabetes at study baseline and participants who were diagnosed with T2D between baseline and follow-up3, we conducted Cox proportional hazard models and adjusted for the same covariates as in the main analysis. Entry time in Cox models was defined as the baseline or follow-up3 time.

The analyses were performed for all single imputation datasets, and the results were combined based on Rubin’s rules. Proportional hazards were assessed with Schoenfeld residuals. All analyses were performed using SAS (version 7.1) and R (version 4.3.0) statistical software.

## 3. Results

Table 1 shows the baseline characteristics of participants (n = 1064) by sex. The median age at diabetes diagnosis was 60 (Q1, Q3: 53–65) years, and 54% were males. A total of 388 (36.5%) participants developed any vascular complications, 82 (7.7%) macrovascular complications and 343 (32.2%) microvascular complications. Baseline characteristics of the sub-sample used for the analysis of change in protein intake from baseline to follow-up3 (n = 663) were similar (Supplementary Table S1).

### 3.1. Protein Intake Prior to Diagnosis and Diabetes-Related Complications

A higher protein intake at the expense of carbohydrates appeared to be associated with a lower risk of complications. However, the association was not statistically significant in any model (HR model 3: 0.83, 95% CI: 0.60–1.14). Relatively similar HRs were observed for animal protein compared to total protein intake. Replacing carbohydrates with an isoenergetic amount of plant protein was also not significantly associated with any complications risk, but estimated HRs had much lower precision (wider 95% CIs) than for total and animal protein (Table 2). Since no significant associations were found when replacing carbohydrates with animal and plant proteins, we conducted a sex-specific analysis only for the substitution of total protein. Sex-specific results were in line with the total sample results, indicating a trend for an inverse association with complications risk when carbohydrates were replaced with total protein (Supplementary Table S2). Substituting plant protein for an isoenergetic amount of animal protein was not statistically significantly associated with any complication (Supplementary Table S3).

### 3.2. Change of Protein Intake from Pre to Post Diabetes Diagnosis and Risk of Complications

Table 3 shows the associations of an isocaloric increase in protein intake (substituting carbohydrates) from pre-diagnosis (study baseline) to post-diagnosis (follow-up3) and complications risk. Replacing carbohydrates with protein was not appreciably associated with total complications and microvascular complications (including nephropathy and neuropathy), e.g., the HR for total complications for a 5% E substitution was 0.98 (95% CI 0.89–1.08) (model 3).

We further evaluated whether these associations depend on the amount of baseline protein intake. Although an increase in protein intake from baseline to follow-up3 was related to lower nephropathy risk in participants with low protein intake at baseline, this association was not statistically significant (HR for 5% E substitution was 0.75; 95% CI: 0.36, 1.54) and also not statistically different from the group with high baseline protein intake (HR for 5% E substitution was 0.92; 95% CI: 0.69, 1.23) (P interaction > 0.005).

We repeated the change analysis considering the follow-up dietary assessment date as the entry time in Cox models instead of the diabetes diagnosis data. These analyses produced overall similar results (Supplementary Table S4).

### 3.3. Protein Intake among Prevalent Diabetes Cases and Diabetes-Related Complications

The analysis for the effect of isoenergetic substitution of total protein (per 5% E) for carbohydrates on myocardial infarction, stroke and total CVD among participants with prevalent diabetes at baseline and participants who were diagnosed between baseline and follow-up3 showed that replacing carbohydrates with an isoenergetic amount of protein was not associated to myocardial infarction, stroke and total CVD risk (Supplementary Table S5). Although not significant, the association between stroke and total CVD was inverse (HRs for 5% E substitution were 0.41; 95% CI: 0.16, 1.01 and 0.64; 95%CI: 0.39, 1.06, respectively) (Supplementary Table S5).

## 4. Discussion

To our knowledge, the present prospective study is among the first investigations to examine the associations of protein intake with vascular complications of T2D in newly diagnosed T2D patients. Our isoenergetic substitution models did not indicate associations between replacing carbohydrates with total, animal and plant protein with T2D complications. Neither considering the pre-diagnosis diet nor the change of diet from pre-diagnosis to post-diagnosis or the post-diagnosis diet revealed any clear association.

Previous studies suggested that total and animal protein intake may increase the risk of incident T2D [1]. In a meta-analysis with 38,350 T2D cases, high total and animal protein intake increased T2D risk (RR 1.10, *p* = 0.006 and RR 1.13, *p* = 0.013), whereas high plant protein intake did not affect T2D risk (RR 0.93, *p* = 0.074) [1]. We found that consuming energy from total, animal as well as plant protein at the expense of energy from carbohydrates was not associated with total and vascular complications of T2D. The mean dietary protein intake in our study population was less than other European diabetes populations (75.7 vs. 91.0–94.2 g/day) [20,21]. This could potentially be indicative of dietary adaptations associated with T2D in previous cohorts, with a particular focus on limiting energy and carbohydrate intake or regional differences in dietary habits. Since our study population included newly diagnosed T2D patients, it is worth noting that it is unlikely that individuals within the study population had received prior dietary guidance specifically targeting protein restriction.

The source of dietary protein (animal versus plant) plays a role in determining both insulin sensitivity and vascular risk. Animal protein consumption triggers glucagon secretion, thereby exacerbating insulin resistance, while plant protein enhances insulin sensitivity [31]. Additionally, consumption of animal protein leads to an elevation in GFR, an increase in albuminuria, and expedites the rate of kidney function deterioration, whereas plant protein has a protective effect on the kidneys [32]. However, we found no statistically significantly increased risk of complications when plant protein was substituted for an isoenergetic amount of animal protein. The reason for the lack of association could be the generally low intake of plant protein in our cohort (mean: 1.2% E). Different plant protein sources may exert heterogeneous relations to health outcomes (e.g., soy-based vs. non–soy-based) [13]. In our cohort, the main food groups contributing to plant protein intake were legumes (beans and peas eaten along with potatoes and meats) and soups.

Although the application of a protein-restricted diet remains controversial in the management of chronic kidney disease [33,34], protein restriction is traditionally used in chronic kidney disease patients [12,35,36], including diabetic nephropathy, since a high-protein diet can worsen glomerular hyperfiltration, which plays a pivotal role in the worsening of kidney function [37]. However, currently, there is no clear evidence regarding protein restriction’s efficacy in attenuating the progression of diabetic nephropathy [37,38,39,40,41]. A study involving 382 patients with T2D from the Diabetes and Lifestyle Cohort Twente (DIALECT) found that unrestricted dietary protein intake (>163 g/day) was not associated with an increased hazard of renal function deterioration (HR 0.42; 95% CI: 0.18–1.00) [20]. Additionally, a meta-analysis of randomised controlled trials indicates that a low-protein diet (a diet with <0.8 g/kg protein) did not show a substantial improvement in renal function markers among patients with either type 1 or type 2 diabetic nephropathy compared to normal/free protein intake, despite a decrease in HbA1c levels and urinary protein excretion [37]. We also found no evidence of a positive association between substituting carbohydrates with protein and the risk of nephropathy, even among participants with higher baseline protein intake levels.

In this study, although there was no statistically significant association between substituting carbohydrates with protein and macrovascular complications risk, estimates of HR in our study pointed towards inverse associations in newly diagnosed participants. Additionally, we found that isoenergetic substitution of total protein for carbohydrates was inversely associated with stroke and total CVD among prevalent cases. In line with our study, previous studies on diabetes complications that focused on prevalent cases also found that high protein intake could be beneficial for preventing CVD risk [40,41]. A previous study involving individuals with T2D who were overweight or obese found that a diet characterised by low glycemic index (25% of the energy intake) and high protein intake (30% of the energy intake) can modulate diastolic dysfunction as well as reduce insulin resistance and potentially delay or prevent the development of diabetic cardiomyopathy [22]. Another study also observed that high protein intake (15–16% of the energy intake) was associated with lower risks of all-cause and CVD mortality in patients with T2D compared to low protein intake (<13% of the energy intake) [23].

This study has several strengths, including its prospective design, the long follow-up, physician-verified endpoints and a high response rate in follow-up for complications. We were able to examine not only diet after diagnosis of diabetes but also pre-diagnosis diet and changes in protein intake over time. The available health, socioeconomic, and nutrition information at both time points (baseline and follow-up) allowed us to control for baseline levels and changes in confounders in the multivariable adjustment models.

The study has some potential limitations. FFQs are generally susceptible to misreporting, e.g., due to social desirability or cognitive challenges in estimating average intake in the previous 12 months. A previous validation study in our cohort indicates that protein intake is underreported by ~20% and moderately correlated with estimates from repeated urinary nitrogen excretion, similar to comparable assessment instruments [27]. Additionally, baseline and follow-up FFQs are not identical, and we cannot rule out that systematic differences in the assessment instruments can have an impact on the estimated intake of food items and nutrients. Unmeasured confounding remains possible, as with all observational studies. We did not have information on metabolic and cardiovascular health markers or other macrovascular complications such as peripheral arterial disease. Incidence of microvascular complications were documented by treating physicians and were not consistently monitored throughout routine follow-up. Nevertheless, in accordance with the National Disease Management Guidelines [42], it is recommended that individuals with diabetes undergo annual screenings for vascular complications in Germany, and their care is primarily overseen by treating physicians. As for a benchmark for our data, we are not aware of a national source in Germany that provides information about the frequency of diabetes-related complications. Lastly, the predominant inclusion of individuals of Caucasian ethnicity with higher socioeconomic status in our study limits the generalizability of the findings to a broader population.

## 5. Conclusions

In summary, a higher intake of total, animal and plant protein at the expense of carbohydrates was not associated with an increased risk of diabetes complications in our cohort. Due to the limited sample size of our study, it is imperative to validate these results through confirmatory analyses involving larger cohort studies with a greater number of endpoints. Furthermore, expanding the research to encompass higher levels of plant protein consumption is essential to comprehensively assess the relationship between plant protein intake and diabetes complications. Controlling for a wide range of confounding variables, extending the follow-up period, and conducting randomised controlled trials will further elucidate the causal link between protein intake and diabetes complications. Such findings will strengthen dietary recommendations for protein to prevent diabetes complications.

## Figures and Tables

**Table 1:** Baseline characteristics of study sample by sex.

Characteristics	Total (n = 1064)	Men (n = 577)	Women (n = 487)
Age at diabetes diagnosis (years) (median (Q1, Q3))	60 (53–65)	60 (53–65)	61 (54–66)
Education (n(%))			
No school degree or primary school	481 (45.2)	223 (38.7)	258 (53.0)
Technical or professional school	267 (25.1)	117 (20.3)	150 (30.8)
University degree	316 (29.7)	237 (41.0)	79 (16.2)
Pre-diagnosis lifestyle			
Physical activity (h/week) (median (Q1, Q3))	1 (0–3.3)	1 (0–3)	1 (0–3.5)
Smoking status (n (%))			
Never-smoker	421 (39.6)	134 (23.2)	287 (59.1)
Former smoker	464 (43.6)	331 (57.3)	133 (27.4)
Current smoker	179 (16.8)	112 (19.5)	67 (13.5)
Smoking duration (years) (median (Q1, Q3))	10 (0, 28)	19 (3, 31)	0 (0–19)
Body mass index (kg/m^2^) (median (Q1, Q3))	29.9 (27.4–33.2)	29.7 (27.5–32.5)	30.2 (27.2–34.0)
Medical information			
Diabetes duration (years) (median (Q1, Q3))	12.1 (9.4–14.9)	12.5 (9.7, 15.1)	11.5 (9–14.6)
Family history of myocardial infarction (n (%))	183 (17.0)	84 (14.5)	99 (20.3)
Family history of stroke (n (%))	219 (21.0)	99 (17.1)	120 (24.7)
Hypertension (n (%))	853 (80.0)	466–467 (80.8)	386 (79.3)
Dyslipidaemia (n (%))	779 (73.2)	422–423 (73.2)	356 (73.1)
Dietary intake (median (Q1, Q3))			
Energy intake (kJ)	8767 (7070–10,661)	9852 (8021–11,574)	7544 (6371–9226)
Protein intake (g/day)	75.7 (60.6–93.4)	85.8 (69.3–101.5)	66.1 (53.6–79.4)
Animal protein intake (g/day)	49.0 (38.4–62.6)	56.3 (43.0–69.5)	43.3 (34.3–53.4)
Plant protein intake (g/day)	25.2 (20.3–31.4)	28.6 (23.5–34.5)	21.8 (18.5–26.8)
Carbohydrate intake (g/day)	222.5 (179.8–274.6)	241.6 (198.4–291.0)	201.8 (166.0–247.5)
Fat intake (g/day)	81.7 (65.1–105.6)	92.7 (71.8–116.8)	72.4 (60.1–90.9)
Alcohol intake (g/day)	9.1 (2.8–21.7)	16.1 (7.5–33.3)	4.2 (1.5–9.4)
Fibre intake (g/d)	21.1 (17.3–26.3)	22.3 (17.9–27.3)	20.0 (16.8–24.0)
PUFA:SFA ratio	0.5 (0.4–0.6)	0.5 (0.4–0.6)	0.5 (0.4–0.5)
Mg intake (mg/d)	0.3 (0.2–0.4)	0.4 (0.3–0.4)	0.3 (0.2–0.3)
Cholesterol intake (mg/d)	0.3 (0.2–0.4)	0.3 (0.2–0.4)	0.3 (0.2–0.3)
Vitamin E intake (mg/d)	11.9 (9.5–14.8)	12.5 (9.8–15.6)	11.3 (9.2–14.0)

Data are presented as median (Q1, Q3) or n (percentage) as applicable. The table presents combined rounded values from the 10 imputation datasets. Percentages may not correspond to anticipated values. Mg: magnesium; PUFA: polyunsaturated fatty acids; SFA: saturated fatty acids.

**Table 2:** HRs and 95% CIs for microvascular and macrovascular complications of type 2 diabetes for isoenergetic substitution (per 5% E) of protein for carbohydrates.

Complications	Total			
	Events (n)	Total Protein	Animal Protein	Plant Protein
		HR (95% CI)	HR (95% CI)	HR (95% CI)
Total complications	388			
Model 1		1.01 (0.78, 1.32)	1.01 (0.78, 1.31)	1.20 (0.53, 2.70)
Model 2		0.89 (0.68, 1.16)	0.89 (0.68, 1.16)	1.02 (0.43, 2.39)
Model 3		0.83 (0.60, 1.14)	0.82 (0.60, 1.13)	1.62 (0.61, 4.27)
Macrovascular complications	82			
Model 1		0.76 (0.42, 1.38)	0.76 (0.42, 1.38)	3.11 (0.40, 16.98)
Model 2		0.81 (0.45, 1.47)	0.82 (0.44, 1.49)	3.36 (0.53, 21.22)
Model 3		0.81 (0.39, 1.68)	0.77 (0.37, 1.61)	3.25 (0.52, 20.01)
Microvascular complications	343			
Model 1		1.09 (0.82, 1.44)	1.09 (0.82, 1.44)	0.90 (0.39, 2.07)
Model 2		0.90 (0.67, 1.21)	0.90 (0.67, 1.22)	0.74 (0.30, 1.79)
Model 3		0.83 (0.58, 1.19)	0.83 (0.58, 1.19)	1.19 (0.41, 3.46)
Nephropathy	200			
Model 1		1.06 (0.72, 1.54)	1.06 (0.72, 1.54)	0.91 (0.34, 2.41)
Model 2		0.85 (0.57, 1.27)	0.85 (0.57, 1.27)	0.84 (0.31, 2.33)
Model 3		1.03 (0.64, 1.64)	0.87 (0.43, 1.76)	1.42 (0.36, 5.55)
Neuropathy	210			
Model 1		1.10 (0.77, 1.57)	1.10 (0.77, 1.57)	0.80 (0.26, 2.44)
Model 2		0.93 (0.64, 1.36)	0.94 (0.65, 1.36)	0.64 (0.20, 2.02)
Model 3		0.76 (0.49, 1.18)	0.77 (0.50, 1.19)	1.15 (0.27, 4.92)

The table presents combined rounded values from the ten imputation datasets. Model 1: age, sex, baseline energy intake, baseline fat intake, baseline alcohol intake. Model 2: Model 1 + education, physical activity, BMI, smoking status and duration, family history of myocardial infarction and stroke, prevalent hypertension and dyslipidaemia, antidiabetic, lipid-lowering and antihypertensive medication, duration between diet assessment and T2D diagnosis. Model 3: Model 2 + baseline fibre intake, PUFA/SFA ratio, baseline cholesterol intake, baseline Mg intake, baseline vitamin E intake.

**Table 3:** HRs and 95% CIs for microvascular and macrovascular complications of type 2 diabetes for isoenergetic increase of protein intake from baseline to follow-up (per 5% E) at the expense of carbohydrates with type 2 diabetes diagnosis date being the entry time (baseline).

Complications	Total		Low Protein Intake		High Protein Intake	
	Events (n)	HR (95% CI)	Events (n)	HR (95% CI)	Events (n)	HR (95% CI)
Total complications	267		126		141	
Model 1		1.00 (0.95, 1.07)		0.97 (0.88, 1.07)		1.06 (0.98, 1.16)
Model 2		1.02 (0.94, 1.10)		0.93 (0.82, 1.04)		1.05 (0.92, 1.21)
Model 3		0.98 (0.89, 1.08)		1.01 (0.82, 1.24)		0.95 (0.80, 1.13)
Microvascular complications	241		116		125	
Model 1		1.03 (0.97, 1.10)		1.02 (0.92, 1.12)		1.08 (0.98, 1.18)
Model 2		1.06 (0.98, 1.16)		0.99 (0.87, 1.13)		1.12 (0.96, 1.30)
Model 3		1.02 (0.92, 1.14)		1.01 (0.81, 1.25)		1.03 (0.86, 1.25)
Nephropathy	148		69		79	
Model 1		1.01 (0.94, 1.09)		0.97 (0.86, 1.09)		1.13 (1.02, 1.26)
Model 2		1.03 (0.82, 1.14)		0.80 (0.61, 1.05)		1.18 (0.96, 1.44)
Model 3		0.93 (0.81, 1.06)		0.75 (0.36, 1.54)		0.92 (0.69, 1.23)
Neuropathy	148		72		76	
Model 1		1.06 (0.97, 1.16)		1.10 (0.94, 1.29)		1.06 (0.95, 1.19)
Model 2		1.01 (0.90, 1.14)		0.98 (0.74, 1.29)		1.04 (0.82, 1.33)
Model 3		0.99 (0.83, 1.17)		1.03 (0.66, 1.60)		0.91 (0.54, 1.53)

The table presents combined rounded values from the ten imputation datasets. Model 1: age, sex, energy intake, baseline protein intake, fat intake (baseline and follow-up), alcohol intake (baseline and follow-up), duration between baseline and diet assessment, duration between T2D diagnosis and diet assessment. Model 2: Model 1 + education, physical activity (baseline and follow-up), BMI (baseline and follow-up), smoking status and duration (baseline and follow-up), family history of myocardial infarction and stroke, prevalent hypertension and dyslipidaemia, antidiabetic, lipid-lowering and antihypertensive medication. Model 3: Model 2 + fibre intake (baseline and follow-up), PUFA/SFA ratio (baseline and follow-up), cholesterol intake (baseline and follow-up), Mg intake (baseline and follow-up), vitamin E intake (baseline and follow-up).

## Data Availability

The datasets analysed during the current study are not publicly available due to data protection regulations. In accordance with German Federal and State data protection regulations, epidemiological data analyses of EPIC-Potsdam may be initiated upon an informal inquiry addressed to the secretariat of the Human Study Center (Office.HSZ@dife.de). Each request will then have to pass a formal application process and be reviewed by the respective PI and a scientific board.

## Supplementary Materials

The following supporting information can be downloaded at: https://www.mdpi.com/article/10.3390/metabo14030172/s1, Table S1: Baseline characteristics of study sample in the protein intake change analysis by protein intake groups at baseline; Table S2: HRs and 95% CIs for microvascular and macrovascular complications of type 2 diabetes for isoenergetic substitution (per 5% E) of protein for carbohydrates by sex; Table S3: HRs and 95% CIs for T2D complications for isoenergetic substitution of animal protein for plant protein (per 5% E); Table S4: HRs and 95% CIs for microvascular and macrovascular complications of type 2 diabetes for isoenergetic increase of protein intake from baseline to follow-up (per 5% E) at the expense of carbohydrates with dietary assessment date being the entry time (baseline); Table S5: HRs and 95% CIs for macrovascular complications for isoenergetic substitution of total protein for carbohydrates (per 5% E) among participants with prevalent type 2 diabetes.
