# Supplementary material for: Association between Protein Intake and Diabetes Complications Risk Following Incident Type 2 Diabetes: The EPIC-Potsdam Study
Source: Metabolites. 2024 Mar 19;14(3):172. doi: 10.3390/metabo14030172 (PMC10972425; doi:10.3390/metabo14030172)
Supplement: Supplementary file 1 [file metabolites-14-00172-s001.zip › metabolites-2906722-supplementary.pdf]

Association between Protein Intake and Diabetes Complications Risk Following Incident Type 2 Diabetes: The EPIC-Potsdam Study

Supplemental Material

Page	Item
2	**Supplementary Table S1.** Baseline characteristics of study sample in the protein intake change analysis by protein intake groups at baseline
3	**Supplementary Table S2.** HRs and 95% CIs for microvascular and macrovascular complications of type 2 diabetes for isoenergetic substitution (per 5% E) of protein for carbohydrates by sex
4	**Supplementary Table S3.** HRs and 95% CIs for T2D complications for isoenergetic substitution of animal protein for plant protein (per 5% E)
5	**Supplementary Table S4**. HRs and 95% CIs for microvascular and macrovascular complications of type 2 diabetes for isoenergetic increase of protein intake from baseline to follow-up (per 5% E) at the expense of carbohydrates with dietary assessment date being the entry time (baseline)
6	**Supplementary Table S5.** HRs and 95% CIs for macrovascular complications for isoenergetic substitution of total protein for carbohydrates (per 5% E) among participants with prevalent type 2 diabetes

RESTRICTED

Supplementary Table S1. Baseline characteristics of study sample in the protein intake change analysis by protein intake groups at baseline

Characteristics	Total (n=663)	Low Protein Intake (n=332)	High Protein Intake (n=331)
Demographics			
Age at diabetes diagnosis (years) (median (Q1, Q3))	59 (53-64)	60 (53-65)	58 (52-63)
Education (n(%))			
No school degree or primary school	296 (44.8)	152 (46.0)	144 (43.4)
Technical or professional school	165 (24.9)	90 (27.1)	75 (22.6)
University degree	202 (30.4)	90 (26.8)	112 (34.0)
Body mass index (kg/m^2) (median (Q1, Q3))	29.9 (27.6-33.2)	29.7 (27.3-32.6)	30.3 (28.1-33.7)
Pre-diagnosis lifestyle			
Physical activity (h/week) (median (Q1, Q3))	1 (0-3)	0.8 (0-2.8)	1.4 (0-3.5)
Smoking status (n (%))			
Never-smoker	247 (37.2)	130 (39.2)	117 (35.2)
Former smoker	303 (45.7)	150 (45.2)	153 (46.1)
Current smoker	113 (17.1)	52 (15.6)	61 (18.7)
Smoking duration (years) (median (Q1, Q3))	13 (0-28)	10 (0-27)	14 (0-28)
Medical information			
Diabetes duration (years) (median (Q1, Q3))	14.2 (12.4-16.0)	14.2 (13.6-16.0)	14.2 (12.4-16.1)
Family history of myocardial infraction (n(%))	113 (17.0)	60 (18.1)	53 (15.9)
Family history of stroke (n(%))	140 (21.0)	73 (22.0)	66 (20.0)
Hypertension (n(%))	534 (80.5)	268 (80.7)	267 (80.4)
Dyslipidaemia (n(%))	475 (71.7)	239 (72.0)	237 (71.3)
Dietary intake (median (Q1, Q3*))*			
Energy intake (kJ)	8,781 (7,232-10,654)	7,298 (6,299-8,181)	10,436 (9,417-12,195)
Protein intake (g/day)	78.1 (63.5-95.1)	63.5 (54.1-70.1)	95.1 (84.8-107.4)
Carbohydrate intake (g/day)	228.1 (187.2-279.0)	199.0 (161.5-230.5)	269.6 (226.1-315.6)
Fat intake (g/day)	78.9 (63.3-102.1)	64.3 (54.4-75.7)	100.2 (83.4-114.7)
Alcohol intake (g/day)	9.1 (3.0-22.4)	7.0 (2.3-16.9)	12.2 (4.8-30.3)
Fibre intake (g/d)	21.4 (17.5-26.4)	18.9 (15.6-22.3)	25.3 (20.8-28.9)
PUFA:SFA ratio	0.5 (0.4-0.6)	0.5 (0.4-0.6)	0.4 (0.4-0.5)
Mg intake (mg/d)	0.3 (0.3-0.4)	0.3 (0.2-0.3)	0.4 (0.3-0.4)
Cholesterol intake (mg/d)	0.3 (0.2-0.4)	0.2 (0.2-0.3)	0.4 (0.3-0.4)
Vitamin E intake (mg/d)	11.1 (8.7-13.8)	9.3 (7.6-11.5)	12.8 (10.9-15.6)

Data are presented as median (interquartile range (Q1, Q3)) or n (percentage) as applicable. Table presents combined rounded values from the 10 imputation datasets. Percentages may not correspond to anticipated values. Low protein intake < 78.1 g/d; High protein intake ≥ 78.1 g/d

Mg: magnesium; PUFA: polyunsaturated fatty acids; SFA: saturated fatty acids.

RESTRICTED

Supplementary Table S2. HRs and 95% CIs for microvascular and macrovascular complications of type 2 diabetes for isoenergetic substitution (per 5% E) of protein for carbohydrates by sex

	Men		Women	
	Events (n)	Total Protein	Events (n)	Total Protein
**Complications**		HR (95% CI)		HR (95% CI)
**Total complications**	240		148	
Model 1		1.04 (0.75, 1.45)		0.99 (0.64, 1.51)
Model 2		0.91 (0.64, 1.30)		0.82 (0.51, 1.33)
Model 3		0.80 (0.51, 1.25)		0.78 (0.43, 1.42)
**Macrovascular complications**	57		25	
Model 1		0.98 (0.50, 1.91)		0.44 (0.12, 1.58)
Model 2		1.01 (0.50, 2.06)		0.42 (0.11, 1.64)
Model 3		0.96 (0.39, 2.36)		-
**Microvascular complications**	205		138	
Model 1		1.10 (0.76, 1.59)		1.09 (0.69, 1.71)
Model 2		0.92 (0.62, 1.36)		0.87 (0.52, 1.48)
Model 3		0.81 (0.49, 1.35)		0.86 (0.47, 1.56)
**Nephropathy**	122		78	
Model 1		1.01 (0.62, 1.62)		1.26 (0.69, 2.33)
Model 2		0.74 (0.43, 1.27)		0.96 (0.46, 1.99)
Model 3		0.91 (0.47,1.80)		1.06 (0.49, 2.32)
**Neuropathy**	118		92	
Model 1		1.02 (0.63, 1.65)		1.10 (0.63, 1.91)
Model 2		0.96 (0.59, 1.58)		0.81 (0.45, 1.49)
Model 3		0.74 (0.38, 1.45)		0.74 (0.38, 1.46)

Table presents combined rounded values from the ten imputation datasets
Model 1: age, baseline energy intake, baseline fat intake, baseline alcohol intake
Model 2: Model 1 + education, physical activity, BMI, smoking status and duration, family history of myocardial infarction and stroke, prevalent hypertension and dyslipidaemia, antidiabetic, lipid lowering and antihypertensive medication, duration between diet assessment and T2D diagnosis
Model 3: Model 2 + baseline fibre intake, PUFA/SFA ratio, baseline cholesterol intake, baseline Mg intake, baseline vitamin E intake

RESTRICTED

Supplementary Table S3. HRs and 95% CIs for T2D complications for isoenergetic substitution of animal protein for plant protein (per 5% E)

	Events (n)	HR (95% CI)
**Total complications**	385	
Model 1		0.84 (0.39, 1.85)
Model 2		0.87 (0.38, 1.98)
Model 3		0.51 (0.20, 1.30)
**Macrovascular complications**	80	
Model 1		0.25 (0.48, 1.25)
Model 2		0.24 (0.04, 1.50)
Model 3		0.24 (0.03, 1.72)
**Microvascular complications**	342	
Model 1		1.21 (0.54, 2.70)
Model 2		0.23 (0.52, 2.89)
Model 3		0.70 (0.26, 1.91)
**Nephropathy**	199	
Model 1		1.15 (0.46, 2.93)
Model 2		1.01 (0.37, 2.72)
Model 3		0.71 (0.22, 2.26)
**Neuropathy**	210	
Model 1		1.38 (0.47, 4.06)
Model 2		1.47 (0.48, 4.55)
Model 3		0.66 (0.17, 2.59)

Table presents combined rounded values from the ten imputation datasets
Model 1: age, sex, baseline energy intake, baseline carbohydrate intake, baseline fat intake, baseline alcohol intake
Model 2: Model 1 + education, physical activity, BMI, smoking status and duration, family history of myocardial infarction and stroke, prevalent hypertension and dyslipidaemia, antidiabetic, lipid lowering and antihypertensive medication, duration between diet assessment and T2D diagnosis
Model 3: Model 2 + baseline fibre intake, PUFA/SFA ratio, baseline cholesterol intake, baseline Mg intake, baseline vitamin E intake

RESTRICTED

Supplementary Table S4. HRs and 95% CIs for microvascular and macrovascular complications of type 2 diabetes for isoenergetic increase of protein intake from baseline to follow-up (per 5% E) at the expense of carbohydrates with dietary assessment date being the entry time (baseline)

	**Total**		**Low Protein Intake**		**High Protein Intake**	
**Complications**	**Events (n)**	**HR (95% CI)**	**Events (n)**	**HR (95% CI)**	**Events (n)**	**HR (95% CI)**
**Total complications**	257		122		135	
Model 1		0.99 (0.93, 1.06)		0.97 (0.88, 1.07)		1.03 (0.94, 1.12)
Model 2		1.01 (0.93, 1.09)		0.93 (0.83, 1.05)		1.02 (0.88, 1.17)
Model 3		0.98 (0.89, 1.08)		1.02 (0.83, 1.25)		0.92 (0.76, 1.11)
**Microvascular complications**	238		114		124	
Model 1		1.02 (0.96, 1.09)		1.01 (0.92, 1.12)		1.04 (0.95, 1.14)
Model 2		1.06 (0.97, 1.16)		0.99 (0.87, 1.12)		1.09 (0.93, 1.28)
Model 3		1.02 (0.92, 1.14)		1.01 (0.81, 1.26)		1.01 (0.84, 1.22)
**Nephropathy**	147		69		78	
Model 1		1.01 (0.94, 1.09)		0.97 (0.86, 1.09)		1.08 (0.96, 1.22)
Model 2		1.02 (0.91, 1.15)		0.80 (0.61, 1.05)		1.10 (0.87, 1.38)
Model 3		0.92 (0.80, 1.05)		0.73 (0.03, 2.06)		0.84 (0.61, 1.15)
**Neuropathy**	147		71		76	
Model 1		1.04 (0.95, 1.14)		1.08 (0.92, 1.27)		1.02 (0.90, 1.15)
Model 2		1.01 (0.90, 1.14)		0.97 (0.74, 1.29)		1.04 (0.82, 1.33)
Model 3		0.98 (0.83, 1.12)		1.03 (0.66, 1.59)		0.91 (0.54, 1.52)

Table presents combined rounded values from the ten imputation datasets
Model 1: age, sex, energy intake, baseline protein intake, fat intake (baseline and follow-up), alcohol intake (baseline and follow-up), duration between baseline and diet assessment, duration between T2D diagnosis and diet assessment
Model 2: Model 1 + education, physical activity (baseline and follow-up), BMI (baseline and follow-up), smoking status and duration (baseline and follow-up), family history of myocardial infarction and stroke, prevalent hypertension and dyslipidaemia, antidiabetic, lipid lowering and antihypertensive medication
Model 3: Model 2 + fibre intake (baseline and follow-up), PUFA/SFA ratio (baseline and follow-up), cholesterol intake (baseline and follow-up), Mg intake (baseline and follow-up), vitamin E intake (baseline and follow-up)

RESTRICTED

Supplementary Table S5. HRs and 95% CIs for macrovascular complications for isoenergetic substitution of total protein for carbohydrates (per 5% E) among participants with prevalent type 2 diabetes

Complications	Events (n)	HR (95% CI)
**Myocardial Infarction**	44	
Model 1		1.09 (0.59, 2.01)
Model 2		1.07 (0.63, 1.80)
Model 3		0.98 (0.46, 2.10)
**Stroke**	52	
Model 1		0.73 (0.39, 1.38)
Model 2		0.83 (0.42, 1.63)
Model 3		0.41 (0.16, 1.01)
**Total Cardiovascular Diseases**	93	
Model 1		0.83 (0.55, 1.25)
Model 2		0.97 (0.64, 1.47)
Model 3		0.64 (0.39, 1.06)

Model 1 = sex, age, energy intake, alcohol intake, fat intake
Model 2 = Model 1 + education, physical activity, BMI, smoking status and duration, family history of myocardial infarction and stroke, antidiabetic, lipid lowering and antihypertensive medication
Model 3 = Model 2 + fibre intake, PUFA/SFA ratio, cholesterol intake, Mg intake, vit E intake
Covariates are based on baseline questionnaire.

RESTRICTED
